# Niche Overlap Is Not Enough: Same Overlap, Contrasting Fluctuations

**DOI:** 10.1111/ele.70425

**Published:** 2026-06-24

**Authors:** Akiva Goldberg, Oshrit Shtossel, Yoram Louzoun, Nadav M. Shnerb

**Affiliations:** ^1^ Department of Physics Bar‐Ilan University Ramat Gan Israel; ^2^ Department of Mathematics Bar‐Ilan University Ramat Gan Israel; ^3^ School of Computer Science College of Management Rishon LeZion Israel

## Abstract

Niche overlap (NO) is a cornerstone of coexistence theory, summarising the strength of competitive coupling among species. Yet NO collapses distinct mechanisms into a single value and may miss key dynamical features. We quantify this limitation by examining temporal correlations in species abundances, a key out‐of‐equilibrium observable in microbial ecology. Using a MacArthur‐type consumer‐resource model, we show that communities with identical NO can display opposite dynamical patterns. Within the resource‐mediated fluctuation regime studied here, a yield‐depletion mismatch (YDM)—the difference between depletion and yield dissimilarities—consistently predicts the sign and magnitude of abundance correlations across analytical approximations, stochastic simulations, and a reanalysis of microbial time series. In contrast, growth‐rate correlations are governed by NO. More broadly, our results show that dynamical observables can depend on mechanistic details beyond those summarised by niche overlap.

## Graphical abstract

Niche overlap summarizes competitive coupling at equilibrium, but it can fail to predict out‐of‐equilibrium dynamics such as temporal abundance correlations. We show that communities with identical niche overlap can exhibit opposite correlation patterns, and that a yield‐depletion mismatch (depletion dissimilarity minus yield dissimilarity) consistently predicts the sign and magnitude of abundance correlations across analytical approximations, stochastic simulations, and reanalysis of microbial time series.

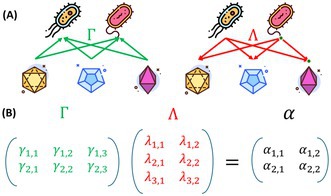

## 1 Introduction

The concept of niche overlap, or conversely, niche segregation, lies at the heart of ecological coexistence theory. As niche overlap between populations increases, so does the extent to which they limit each other's abundance, reducing mutual invasion rates and making coexistence increasingly dependent on compensating fitness differences (Chesson 1994, 2000). Consequently, considerable effort has been devoted to defining niche overlap within theoretical models and quantifying it in empirical and experimental systems (Spaak and De Laender 2020; Schlechter et al. 2023). Beyond its central role in coexistence theory, quantifying niche overlap also enables the partitioning of a diverse community into functional groups, which can facilitate the interpretation and analysis of community dynamics (Crocker et al. 2025; Giral Martínez et al. 2025).

Despite its key role in coexistence theory, it remains unclear how far niche overlap (NO) can constrain the dynamical properties of ecological communities, even when fitness differences are absent. NO is a composite equilibrium summary of competitive coupling, and distinct mechanistic configurations can yield the same NO value. As a result, communities matched for NO may nevertheless exhibit different transient and fluctuation‐driven responses to perturbations. This raises the question of which out‐of‐equilibrium quantities are fixed by NO, and which require additional mechanistic parameters (West and Shnerb 2022).

One of the most accessible and analytically tractable manifestations of out‐of‐equilibrium dynamics is the pattern of correlations between species' abundances over time. Such correlations quantify the extent to which fluctuations in one species' abundance are mirrored by fluctuations in another's, and they can be estimated from time‐series data. Because these correlations determine the degree of synchrony among species, they are closely connected to community stability (Loreau and De Mazancourt 2013). Abundance correlations have become especially prominent in microbial community studies due to the growing availability of high‐resolution time series (Crocker et al. 2025; Goyal et al. 2022; Sireci et al. 2023). In this context, correlations are often interpreted as indicators of niche overlap, under the assumption that species with greater overlap will display stronger correlations.

However, even a priori, abundance correlations reveal a limitation of niche overlap (NO) as a sole descriptor. Although correlations tend to increase with NO (Crocker et al. 2025), the *sign* of the effect is intrinsically ambiguous: higher overlap can align species' responses to environmental fluctuations (favouring positive correlations (Sireci et al. 2023)), while simultaneously strengthening competitive coupling, favouring negative correlations via compensatory dynamics.

To address this conundrum, we study a consumer‐resource (CR) system (Sakarchi and Germain 2025), in which species interact indirectly through shared resources. We ask whether temporal abundance correlations are determined by NO (or, more generally, by the effective interaction matrix) alone, or whether additional features of CR dynamics—that is, parameters not captured by the interaction matrix—play a central role. We therefore focus on fluctuations that are transmitted through the consumer‐resource pathways that define niche overlap, while keeping niche overlap itself time invariant. Correlations imposed by direct environmental forcing on species' demographic rates (Sireci et al. 2023) or on their resource uptake traits (Li and Chesson 2016), are outside the scope of the present analysis. We emphasise that we do not analyse fluctuations as coexistence‐stabilising mechanisms (e.g., the storage effect or relative nonlinearity (Chesson 1994, 2000; Chesson and Huntly 1997)). Instead, we restrict attention to parameter regimes with deterministic stable coexistence and analyse fluctuations in the vicinity of the coexistence equilibrium.

In a consumer‐resource system, each consumer species is characterised by two distinct functional profiles. Its *yield* quantifies the growth benefit it gains from one unit of resource, that is, how efficiently resource uptake translates into population growth. Its *depletion* measures the rate at which it consumes or removes each resource per unit of its own biomass. At equilibrium, or when resource dynamics are much faster than the consumer dynamics, these two profiles combine to produce the effective competition coefficients between consumers, and hence the niche overlap (NO). Crucially, the mapping from the full set of CR parameters to the reduced (consumer‐consumer) competition matrix is highly redundant: many distinct CR configurations yield the same NO and the same equilibrium state, yet they may differ in their out‐of‐equilibrium behaviour. This redundancy enables keeping NO fixed while systematically varying the underlying yield and depletion traits.

The conflicting expectations that arise from NO motivate the search for an alternative predictor. Here we introduce the *yield‐depletion mismatch* (YDM), denoted D, which quantifies whether differences between two species in *how they deplete resources* align with differences in *how they benefit from those resources*. *D* = 0 when resource losses are proportional to consumer gains, D differs from zero when depletion and benefit are misaligned. Such a decoupling may reflect differences in conversion efficiency, wasteful or destructive uptake, metabolic by‐products, cross‐feeding and related mechanisms. Indeed, recent studies increasingly suggest that the yield‐depletion ratio is not fixed but varies across conditions, resources and taxa (Liu et al. 2024; Gibbs et al. 2022; Blumenthal et al. 2024). In Supporting Information D2, we illustrate this variability in the yield‐to‐depletion ratio using the monoculture measurements from (Crocker et al. 2025).

The work below combines analytical calculations, stochastic simulations, and a reanalysis of microbial time‐series data (Crocker et al. 2025). Across all three, we find that, indeed, the YDM determines both the sign and magnitude of temporal abundance correlations, whereas niche overlap alone does not predict these patterns.

## 2 Methods

### 2.1 Model Framework

To isolate how niche overlap shapes temporal correlations, we use a consumer‐resource model in which stochasticity enters through resource dynamics while the effective consumer‐consumer interaction matrix (and hence NO) remains time‐invariant. To this end, we implement a slight modification of MacArthur's consumer‐resource model with S consumers and Q resources. The instantaneous abundance of consumer i is denoted by $n_{i}\left(t\right)$, and the available biomass of resource k by $R_{k}\left(t\right)$. Their dynamic satisfies,
(1)

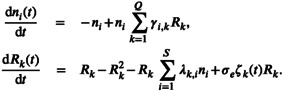

Here $\gamma_{i,k}$ is the $\left\{i,k\right\}$ element of the S×Q matrix Γ that quantifies the yield (the increase in the growth rate of consumer i per unit of resource k), whereas $\lambda_{k,i}$, the elements of a Q×S matrix Λ, quantify depletion (the per‐capita removal of resource k by consumer i). The parameter $\sigma_{e}$ controls stochastic variations in the resource growth rates: $\zeta_{k}\left(t\right)$ denotes Gaussian white noise, formally the time‐derivative of a Wiener process, $\zeta_{k}\left(t\right)=dW_{k}/dt$, with independent $W_{k}\left(t\right)$. We interpret the stochastic term in the *Stratonovich* sense (see Supporting Information B1).

When the dynamics are deterministic (i.e., $\sigma_{e}=0$), the system may converge to a stable equilibrium. In that case, imposing $dR_{k}/dt=0$ allows one to eliminate the resource variables by solving for $R_{k}$ as functions of $\left\{n_{i}\right\}$ (see Supporting Information A). Substituting these expressions back into the consumer equations yields an effective Lotka‐Volterra dynamics,

(2) $$\frac{dn_{i}\left(t\right)}{dt}=n_{i}-n_{i}^{2}-n_{i}\sum_{j\neq i}^{S}\alpha_{i,j}n_{j}.$$

**FIGURE 1:**
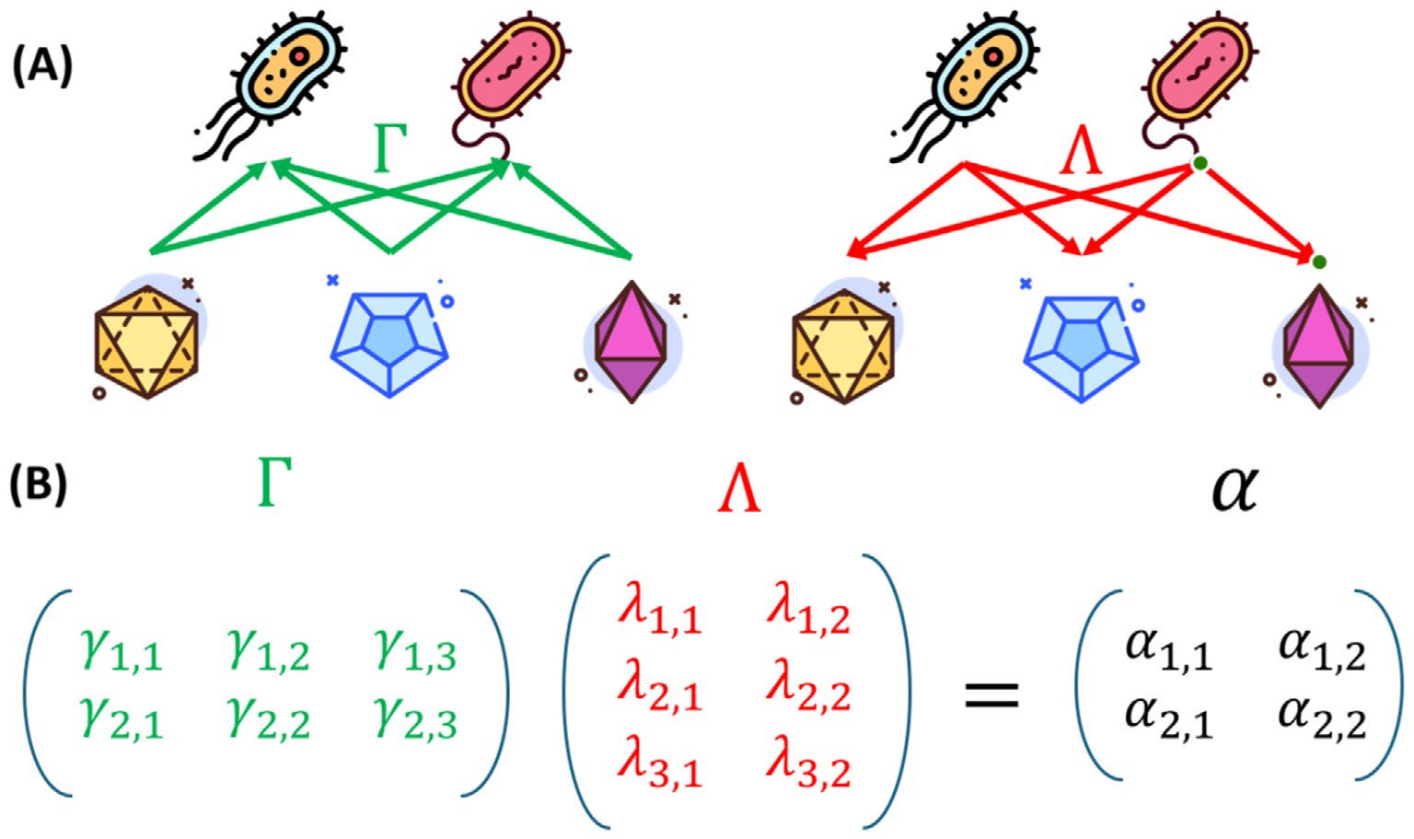
The consumer‐resource model, Equation (1), describes the dynamics of S species and Q resources. Panel (A) illustrates the case in which S=2 and Q=3. Each element of the Γ matrix, $\gamma_{i,k}$, describes the yield (growth rate per unit mass) of the i‐th species due to the k resource (green arrows). Each element of the Λ matrix, $\lambda_{k,i}$, quantifies the depletion of the k resource due to the presence of the i‐th consumer species. Panel (B) illustrates the mapping of the consumer‐resource model (1) into a Lotka‐Volterra model for the consumer species when the nutrient dynamic is fast, so one imposes $dR_{k}/dt=0$. To get Equation (2) in that specific parametrisation each row in the Γ matrix must sum up to 2, and then the α matrix is obtained as the product of Γ and Λ. The mapping from mechanistic parameters $\left(\Gamma ,\Lambda \right)$ to the effective interaction matrix (and thus to niche overlap) is highly redundant, enabling comparisons between distinct consumer‐resource mechanisms that share the same niche overlap.

This mapping holds provided that each row of Γ sums to 2, and that the effective interaction matrix satisfies α=ΓΛ with $\alpha_{i,i}=1$ (see Figure 1).

In this reduced form, all species share the same intrinsic growth rate (set to one), and in the absence of competition ($\alpha_{i,j}=0$ for all i≠j) they also share the same carrying capacity (set to one); the latter property can always be achieved by rescaling species densities (Chesson 2018). In the classical two‐species Lotka‐Volterra setting, niche overlap between species 1 and 2 is given by $\rho_{1,2}=\sqrt{\alpha_{12}\alpha_{21}/\left(\alpha_{11}\alpha_{22}\right)}$ (Chesson 2012). In our parameterisation, the interaction matrix is symmetric with unit diagonal, so $\alpha_{12}$ coincides with $\rho_{1,2}$ and directly represents niche overlap (Spaak and De Laender 2020).

In this parameterisation, the consumer‐resource model has 2SQ parameters (the two matrices γ and λ), which are constrained by $S^{2}+2S$ conditions (the matrix α and the row sum of Γ). Therefore, as explained in detail in Supporting Information A4, the mapping is highly redundant, with many CR systems giving rise to the same Lotka‐Volterra system. We exploit this redundancy to investigate how consumer abundance correlations vary across consumer‐resource systems that share the same α, and to examine how such correlations depend on the underlying mechanistic processes that are not captured by the effective interaction coefficients alone. The core numerical task is to generate the two matrices randomly, up to the imposed constraints. In Supporting Information A5 we detail the two sampling strategies employed in this work.

### 2.2 Calculating Correlations

We employed three complementary methods to calculate abundance and growth‐rate correlations between pairs of species. First, we directly integrated the stochastic differential Equation (1) numerically, tracking abundance and growth rates over time. Second, we identified the deterministic fixed point and estimated the expected correlations using linear response theory, based on the Jacobian matrix at equilibrium and the noise covariance structure, in a manner similar to the stochastic stability analysis developed in Arnoldi et al. (2016). This method is semi‐analytical, as it requires numerical inversion of the Jacobian matrix. Finally, in specific cases involving two species and two resources with carefully chosen interaction coefficients, we were able to solve the system analytically and obtain exact expressions for the correlations. These analytical results guided us in formulating an informed conjecture for a general metric that predicts the sign and magnitude of abundance correlations. The technical details of all three methods are provided in Supporting Information B and C.

### 2.3 Empirical Data Analysis

We reanalysed the dataset from Crocker et al. (2025), who monitored the abundance dynamics of a 20‐strain synthetic microbial community over nine batch‐growth transfer cycles in 32 distinct environments, each defined by a unique mixture of carbon sources. Raw abundance time‐series and monoculture measurements of growth rate and resource depletion were obtained from the publicly available dataset provided by the authors.

Based on these data, we computed the functional dissimilarities in yield and depletion and evaluated the temporal correlation between abundance time series for each pair of strains. Specifically, within each environment we calculated Spearman's rank correlation across time (transfer cycles), and then averaged these correlation values across all environments in which both strains were sufficiently abundant. These empirical correlations were then compared to theoretical predictions based on the YDM and to another parameter $D_{T}$, which, similar to niche overlap, captures the total functional dissimilarity as explained below.

## 3 Results

In this section we first introduce the yield‐depletion mismatch (YDM) metric D and provide its mechanistic interpretation. We then present numerical results showing that D predicts the sign and magnitude of temporal abundance correlations across a broad range of consumer‐resource configurations with fixed niche overlap. Next, we provide empirical support using microbial time‐series data. Finally, we comment on the time‐averaged neutral limit and on the distinct behaviour of correlations in instantaneous growth rates.

### 3.1 The Yield‐Depletion Mismatch Parameter D

As defined above and illustrated in Figure 1, for two species i and j the niche overlap $\alpha_{i,j}$ is given by the inner product of the yield profile of species i, $\gamma_{i,k}$, and the depletion profile of species j, $\lambda_{k,j}$. Under our normalisation, the diagonal elements of α satisfy $\alpha_{i,i}=1$, and if $\gamma_{i,k}=\gamma_{j,k}$ and $\lambda_{k,i}=\lambda_{k,j}$ then $\alpha_{i,j}$ is also 1. Thus, $\alpha_{i,j}$ provides a metric for the overall functional similarity between species.

Now we would like to distinguish between two aspects of functional dissimilarity, namely yield dissimilarity Δγ and depletion dissimilarity Δλ, defined through the cosine distance between the relevant rows (of Γ) or columns (of Λ)

$$\begin{array}{c} \Delta \gamma =1-\cos\theta_{i,j}\left(\gamma \right)\equiv 1-\frac{{\vec{\gamma }}_{\;i}\cdot {\vec{\gamma }}_{\;j}}{|{\vec{\gamma }}_{\;i}\|{\vec{\gamma }}_{\;j}|}\\ \Delta \lambda =1-\cos\theta_{i,j}\left(\lambda \right)\equiv 1-\frac{{\vec{\lambda }}_{i}\cdot {\vec{\lambda }}_{j}}{|{\vec{\lambda }}_{i}\|{\vec{\lambda }}_{j}|}. \end{array}$$

**FIGURE 2:**
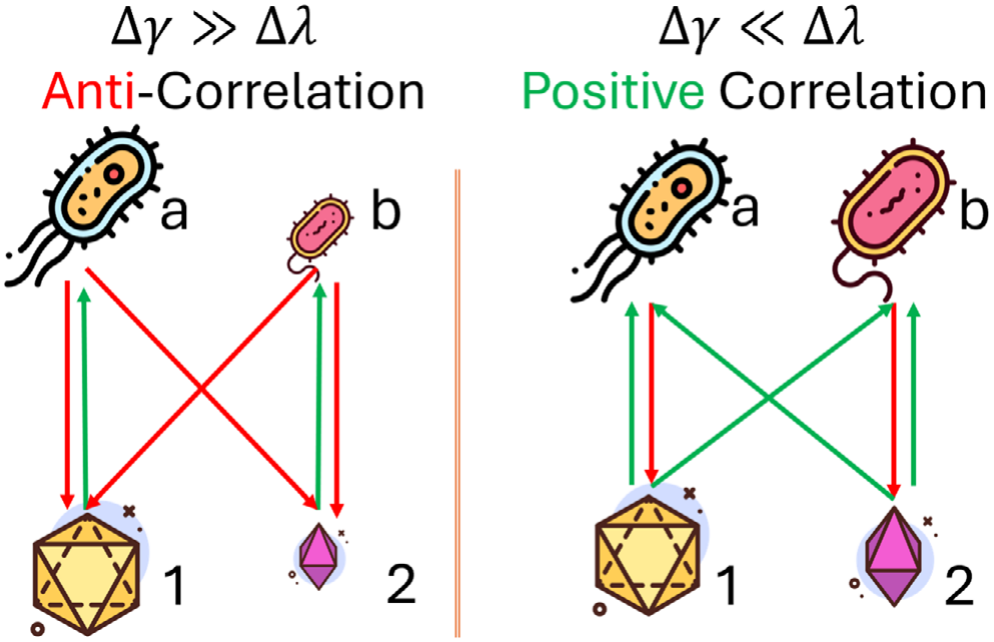
Intuition Behind the Yield‐Depletion Mismatch D. Two schematic examples corresponding to limiting cases. Green lines represent yield; red lines represent depletion. Left: Δγ≫Δλ, that is, each consumer depends on a different resource (green arrows) but depletes (red arrows) both in a similar way. An increase in one resource strongly benefits only one species; since depletion is not species‐specific, this harms the other species, producing negative correlations. Right: Δγ≪Δλ. Now, both consumers are generalists and respond similarly to increases in any resource. Although their depletion profiles differ, the shared response to environmental variation dominates, producing positive correlations. These panels are intentionally stylised and are not meant to represent realistic communities. Natural systems are unlikely to exhibit perfectly generalist or perfectly specialist extremes; instead, yield‐depletion asymmetries may arise from species‐specific processing, wasteful or destructive uptake, cross‐feeding and related processes. The panels aim to clarify the mechanisms by which such asymmetries generate positive or negative abundance correlations.

The sum of these two quantities, $D_{T}\equiv \Delta \gamma +\Delta \lambda$, again reflects the overall functional dissimilarity and thus is a proxy for 1−α. In what follows, though, we will focus mainly on the *yield‐depletion mismatch* D, defined by the *difference* between these two quantities,

D≡Δλ−Δγ,

Focusing on D amounts to the hypothesis that the key determinant of temporal abundance correlations is not the total functional dissimilarity between species, but rather the *imbalance* between yield and depletion dissimilarities. The logic behind this claim is illustrated in Figure 2: differences in yield tend to generate negative correlations, whereas differences in depletion tend to generate positive correlations. We thus expect that even among species pairs with identical niche overlap, correlations will be positive when depletion dissimilarity is dominant, and negative when yield dissimilarity is dominant.

Among the many possible definitions of a yield–depletion mismatch, the specific metric D is a *theory‐guided conjecture* grounded in an exact solution of a tractable two‐species/two‐resource case. In that solvable setting (see Supporting Information C), D captures the overall shape of the correlation, including its zero‐crossings and peak location. Using cosine dissimilarities makes D scale‐free: it depends on the *directions* of the yield and depletion vectors, not on their magnitudes, and Figure 2 suggests that abundance correlations are controlled by relative profiles. Accordingly, rescaling these vectors or swapping the species labels in depletion changes only timescales, or which species responds more, but not the mismatch quantified by D.

**FIGURE 3:**
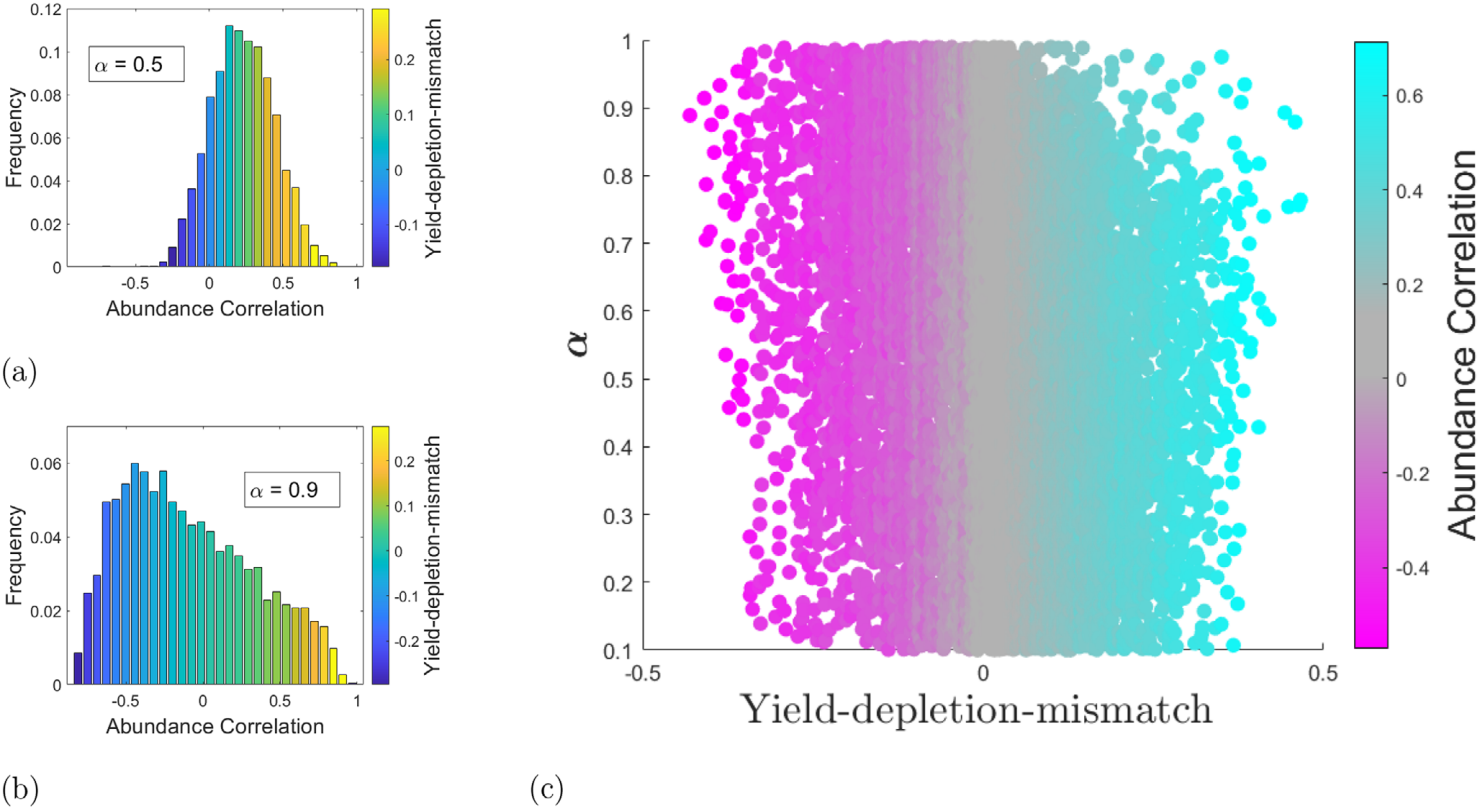
Inadequacy of niche overlap α, and effectiveness of the yield‐depletion mismatch (YDM) D, as predictors of abundance correlations. The histograms in panels (a) and (b) show the frequency of abundance correlations between two consumer species for many realisations with α=0.5 (a) and α=0.9 (b). The colour of each bar indicates the mean value of D in that bin. We observe that α alone is a poor predictor of the correlation, whereas the YDM D captures it well. Panel (c) illustrates this result in a more general setting: Here, colour indicates the level of correlation obtained from numerical experiments across the D–α plane. Clearly, correlations are a function of D, not of α, although the relative frequency of negative D values in the sample space increases as α→1. We first fixed a specific α in a two‐consumer, three‐resource model, and then used the redundancy of the consumer‐resource model to generate many randomly chosen Γ and Λ matrices that yield the same α, as described in Supporting Information A. For each realisation, we computed the correlation between the abundances of the two consumer species, with stochasticity entering through the resource dynamics as described in Equation (1).

Our numerical experiments reveal that D is indeed strikingly predictive, consistently recovers the salient features of abundance correlations without additional fitting. Figure 3 illustrates this agreement for a system with two species and three resources: predictions based on D closely track the measured correlations across parameter sweeps and model variants, underscoring D's practical value as a compact, mechanistic predictor. In contrast, niche overlap α provides very little information about abundance correlations.

**FIGURE 4:**
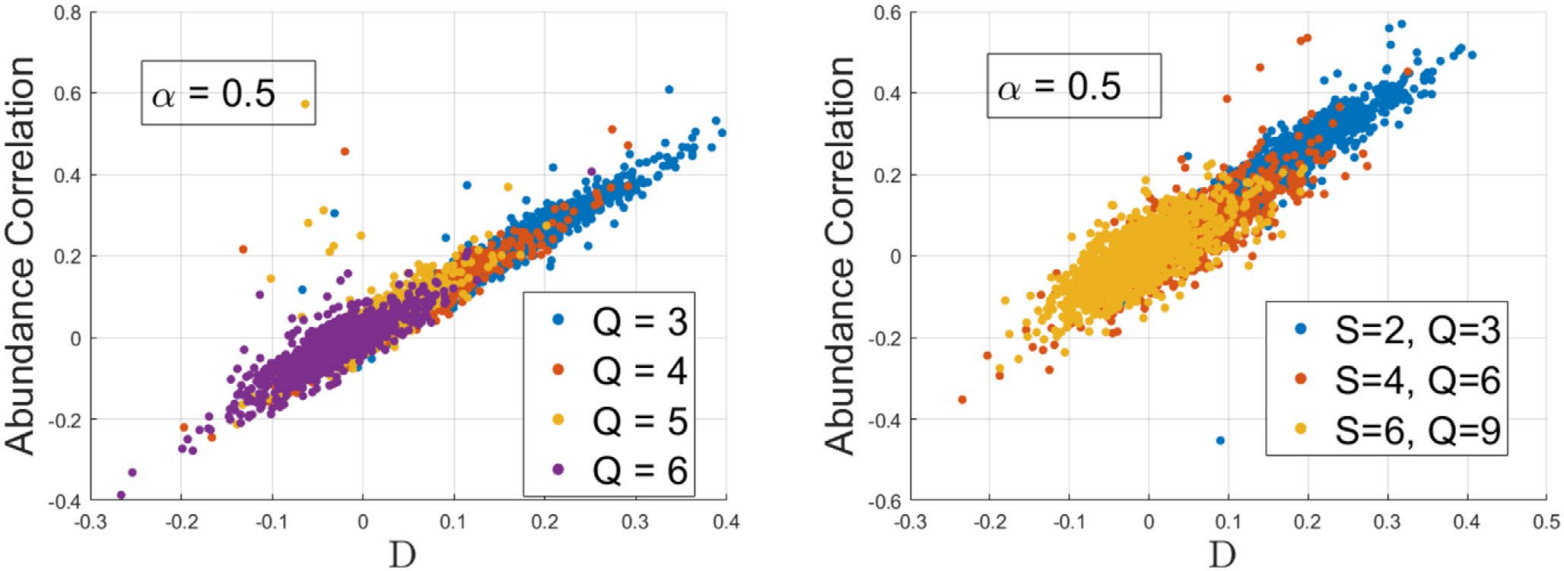
Effect of more resources and more species The left panel illustrates the correlations versus D for a fixed value of the niche overlap α=0.5, in a two‐species model with a varying number of resources. Clearly, the credibility of the YDM parameter D as a predictor of correlations is not affected by the number of resources. The right panel shows the same outcomes for communities with different numbers of species and different numbers of resources.

In Figure 4, we show that D is applicable as a predictor of correlations even when the number of resources is increased, or when the community includes many consumer species. Note that we considered only pairs of species for which the deterministic dynamics allow for a stable coexistence. Other cases, such as those in which the system transitions to chaotic dynamics (Blumenthal et al. 2024; Dalmedigos and Bunin 2020), or cases involving transient species that persist solely due to immigration, require a separate discussion.

### 3.2 Empirical Support

Empirical validation of these theoretical results requires abundance data for several competing species collected in a controlled laboratory setting, where macroscopic conditions such as temperature and pH are kept approximately constant, while metabolite concentrations can fluctuate due to growth and consumption dynamics. In addition, monoculture assays are needed to determine each species' yield and depletion rate per metabolite.

While such comprehensive datasets are rare, a recent study provides a close approximation. Crocker et al. (2025) tracked the temporal dynamics of a synthetic community composed of 20 microbial strains. Each of these strains was first grown in monoculture on one of 10 possible carbon sources (glucose, glycerol, etc.), and its growth rate and its depletion level were measured (see Supporting Information D). Subsequently, the full community was introduced into an environment containing a random mixture of these 10 carbon sources and subjected to 9 batch growth cycles, with a 10‐fold dilution into fresh media (with the same carbon composition) at the end of each cycle. A total of 32 such experiments were conducted, each with a different carbon‐source composition, yielding a dataset of species' abundances for 20 strains over 9 transfer cycles across 32 environments.

For each pair of species, and for each carbon‐source composition (i.e., each environment), we thus have 9 simultaneous abundance measurements and can compute the correlation between the corresponding time series. Although metabolite concentrations are largely reset after each dilution, fluctuations do occur from batch to batch. Moreover, there are also changes in metabolite levels during each growth cycle due to consumption by other species, so it is reasonable to expect that at least part of the observed correlation reflects fluctuations in metabolite availability. Any remaining unexplained component of the observed correlations may nonetheless reflect residual environmental variability, such as temperature fluctuations, that inevitably enters laboratory experiments.

We analysed the dataset described above by computing, for each pair of species, the mean abundance correlation across all environments and examined its dependence on two functional dissimilarity metrics: the yield‐depletion mismatch D=Δλ−Δγ, and the total functional dissimilarity $D_{T}=\Delta \lambda +\Delta \gamma$. The technical reasons why we have chosen the proxy $D_{T}$ over the niche‐overlap parameter α are explained in Supporting Information D3, together with the remaining details of the analysis.

**FIGURE 5:**
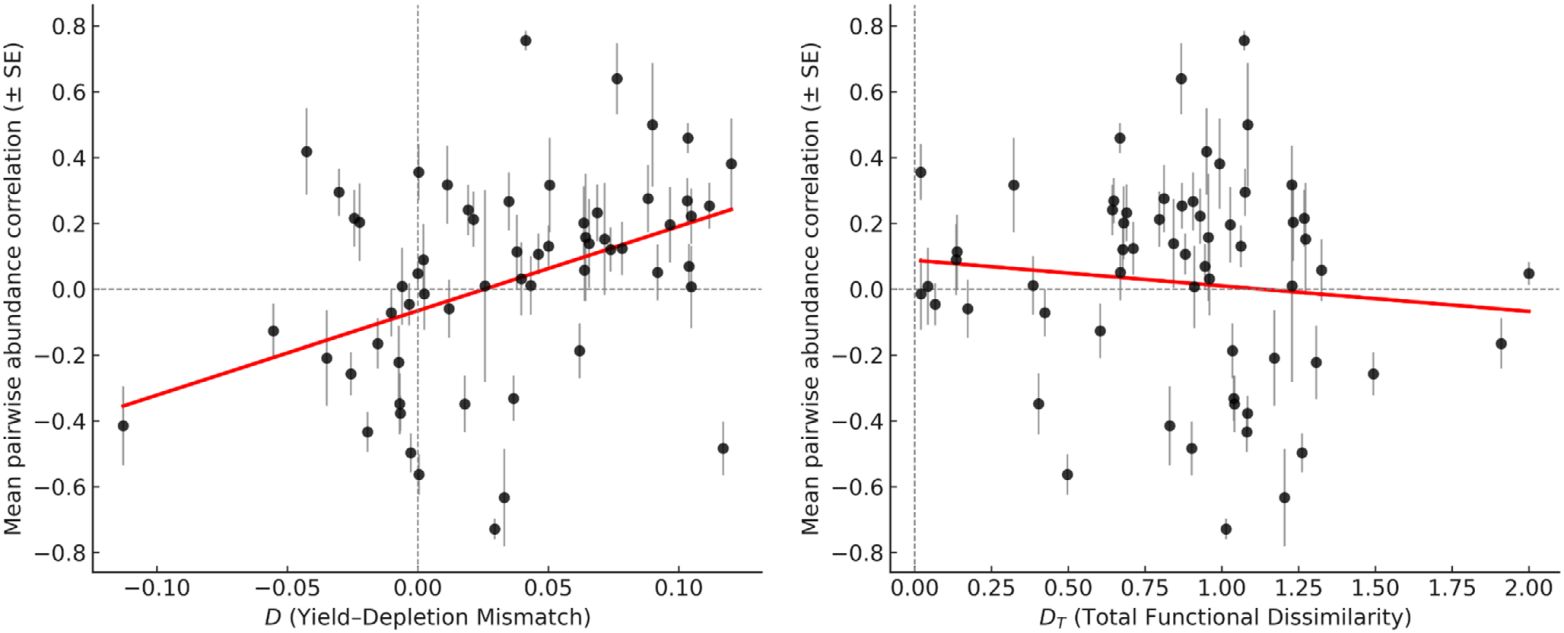
Abundance correlations in empirical community. Abundance correlations between microbial strains as a function of the YDM parameter D (left) and the total functional dissimilarity $D_{T}$ (right). In the left panel, the upward trend is statistically significant with p=0.005. In contrast, in the right panel we find no evidence for a systematic association (p=0.46). To avoid the influence of inferior species approaching extinction, we included in the analysis only the ten most abundant species in each environment. To obtain meaningful confidence intervals, we further restricted the analysis to species pairs that co‐occurred among the top ten in at least three environments. In addition, we considered only data from the second day onward to minimise the effect of transients. Statistical robustness checks of our results are presented in Supporting Information D.

As shown in Figure 5, a statistically significant positive trend is observed between the correlation and D, whereas the trend with $D_{T}$ is weak and not significant. This suggests that the asymmetry between depletion and yield, rather than the total functional dissimilarity, governs co‐abundance patterns, in line with our theoretical framework.

### 3.3 Growth‐Rate Correlations and the Time‐Averaged Neutral Limit

Before concluding this section, we would like to add two brief comments

- The time‐averaged neutral limit: As the system becomes nearly neutral on average (i.e., as α→1), Figure 2 indicates that it becomes increasingly difficult to find Γ and Λ matrices for which D takes positive values. This trend is not an artefact of our sampling procedure, and becomes more pronounced when the mean correlation is plotted against α, see Supporting Information A6. Consequently, in the almost‐neutral limit α→1, two species usually exhibit near‐perfect anti‐correlation. This observation is relevant to many recent works on time‐averaged neutral dynamics (Kalyuzhny et al. 2015; Danino and Shnerb 2018; van Nes et al. 2024; Mallmin et al. 2024).
- Growth‐rate correlations: While abundance correlations are poor indicators of niche overlap, correlations in instantaneous growth rates provide a much better proxy, as shown in Supporting Information A6 (Figure A1). Competitive effects contribute relatively little to growth‐rate correlations, whereas the shared response to environmental fluctuations dominates. At first glance, this may seem surprising, since abundance dynamics are driven by instantaneous growth rates. Yet there is no mathematical inconsistency: a trajectory is indeed the time integral of its derivative, but correlations need not be preserved under differentiation or integration. Consequently, two species can exhibit positively correlated abundances while their instantaneous growth rates are negatively correlated or vice versa.

## 4 Discussion

### 4.1 Biological Origins of Yield‐Depletion Asymmetry

The use of correlations to identify shared dynamical patterns is widespread, despite criticism that has been raised against it in the past (Carr et al. 2019; Fisher and Mehta 2014). As we have seen throughout this paper, abundance correlations between coexisting species do not reflect niche overlap, but rather the balance between shared responses to environmental fluctuations (which promotes positive correlations) and competition (which promotes negative correlations). In cases where the niche structure involves resource partitioning, as in the model considered here, the key quantity is the difference between the depletion gap Δλ and the yield gap Δγ.

In our analysis, we assumed that the matrices Γ and Λ are not necessarily equal, and therefore sampled their entries as entirely independent up to the global constraint on the value of NO. By contrast, in the paper of Sireci et al. (2023) mentioned above, the authors analysed the same consumer‐resource dynamics but assumed that $\gamma_{i,j}=\lambda_{i,j}$ up to a multiplicative factor. As a result, that study covered only the case D=0, and accordingly, no correlations in consumer abundances were found. Sireci et al. (2023) thus had to conclude that the empirically observed correlations arise from shared responses to environmental factors external to the consumer‐resource system, such as temperature, pH, and similar variables. If correlations are dominated by such external drivers, there is no reason to expect a systematic relationship between abundance correlations and niche overlap as inferred from resource use, because even species with very similar resource niches may respond differently to pathogens or temperature. Our work suggests an alternative interpretation of these results: they can be explained within the consumer‐resource framework, provided that genetic distance between microbial strains primarily reflects depletion differences rather than yield differences.

Indeed, recent studies have increasingly adopted the assumption that the ratio between yield and depletion is not fixed (Crocker et al. 2025; Liu et al. 2024; Gibbs et al. 2022; Blumenthal et al. 2024). This assumption is biologically plausible for several reasons. Even within a single species, the yield‐to‐depletion ratio can vary substantially with environmental and physiological conditions, such as oxygen availability (aerobic vs. anaerobic metabolism (Postma et al. 1989)), temperature (Bjørge et al. 2018), and metabolic state (Basan et al. 2015). Across taxa, physiological strategies also differ: ectotherms like insects and fish often achieve higher conversion efficiency than endotherms like mammals, which invest more energy in maintenance and thermoregulation (Speakman 2005). Moreover, organisms sometimes deplete resources without direct benefit, for instance, surplus killing by predators (Vucetich et al. 2002), destructive foraging by elephants (Owen‐Smith 1988), scatter hoarding by rodents (Jansen et al. 2004), or territorial scent‐marking (Gosling and Roberts 2001). While some correlation between yield and depletion is expected, they are unlikely to be identical. Realistic ecological scenarios likely fall between the limiting case analysed by Sireci et al. (2023) and the more general framework considered here. Crucially, once symmetry between yield and depletion is broken, nontrivial correlation patterns emerge.

Furthermore, the empirical data from Crocker et al. (2025) showed that the yield and depletion profiles are not simply related (see Supporting Information D), and this lack of alignment is reflected in the presence of nontrivial abundance correlations, which vanish in the special case D=0.

We focused on the case where the Lotka‐Volterra dynamics emerging from the consumer‐resource equations involve symmetric interactions, that is, $\alpha_{i,j}=\alpha_{j,i}$. We have also analysed the two‐species case with asymmetric interactions ($\alpha_{i,j}\neq \alpha_{j,i}$) and obtained qualitatively similar results. Note that asymmetric Lotka‐Volterra dynamics can arise from an underlying consumer‐resource system only when Γ≠Λ. In diverse communities, such asymmetry may promote chaotic dynamics with no deterministic steady state, as discussed in Gibbs et al. (2022); Blumenthal et al. (2024).

### 4.2 Implications for Inference and Stochastic Modelling

From a broader theoretical perspective, our findings highlight a conceptual separation between the parameters encoded in the reduced competition matrix that govern coexistence and the parameters which control the community's transient and stochastic dynamics. Niche overlap belongs to the former category, whereas the yield‐depletion mismatch is an example of the latter. This distinction explains why NO alone is insufficient to predict abundance correlations and, more generally, why static coexistence metrics may fail to capture dynamical responses (West and Shnerb 2022).

From an empirical standpoint, these results call for caution in interpreting abundance correlations as direct indicators of niche overlap. While such correlations have been widely used for this purpose, we find that they are strongly influenced by YDM and may bear little relation to NO itself. For practitioners aiming to infer NO from data, our analysis indicates that correlations in instantaneous growth rates, rather than abundances, provide a more reliable proxy, as they more directly reflect shared environmental responses without the delayed confounding effects introduced by competition.

Although our work focuses on abundance correlations, its logic suggests that other out‐of‐equilibrium properties may likewise depend on parameters not encoded in NO. Testing these predictions will require both theoretical extensions and empirical measurements in diverse systems.

Finally, we comment on the implications of our approach for modelling species‐rich communities. Such systems are often modelled using deterministic Lotka‐Volterra equations (Kessler and Shnerb 2015; Barbier et al. 2018), which naturally raises the question of how to incorporate environmental stochasticity.

A tempting idea is to tie the correlation structure of the stochastic forcing to niche overlap: functionally similar species should respond more similarly to environmental variability. However, this cannot be implemented in general within an effective Lotka‐Volterra description. Prescribing the correlation matrix of S stochastic time series to be α would require a Cholesky decomposition (Golub and Van Loan 2013), which in turn requires α to be positive semidefinite—a condition that generic ecological interaction matrices do not satisfy. Our framework therefore provides a basis for more realistic stochastic modelling of species‐rich systems, and we hope to develop this direction further in future work.

## Author Contributions

A.G. and N.M.S. conceived the study, developed the theoretical framework, and performed all simulations and analyses. O.S. and Y.L. contributed to interpretation, discussion, and manuscript refinement through significant conceptual suggestions. All authors reviewed and approved the final manuscript.

## Funding

This work was supported by Israel Ministry of Science (Italy‐Israel cooperation) (Grant 7578) and Israel Science Foundation (Grant 2435/24).

## Supporting information


**Supporting Information: A.** From consumer‐resource to Lotka‐Volterra.
**Figure A1:** Correlations versus niche overlap.
**Supporting Information: B** Calculating correlations.
**Figure B1:** Comparison of numerical (X) and analytical (O) correlations across 100 independent trials. Top: abundance correlations *CR*(*n*_1_, *n*_2_). Bottom: growth‐rate correlations *CR*(*r*_1_, *r*_2_). The close alignment across trials demonstrates the accuracy of the theoretical predictions.
**Supporting Information: C** The Yield‐Depletion Mismatch (YDM) metric.
**Figure C1:** Comparison of the abundance correlation cor(λ) and the YDM parameter *D*(λ). Both functions exhibit the same qualitative shape, share the same peak value, and cross zero at the same points.
**Supporting Information: D** Empirical Results.
**Figure D1:** Time series of relative abundances (fraction of the total community) across nine transfer cycles for 20 microbial strains in one environment (a fixed carbon mixture).
**Figure D2:** The ratio *C* = λ_k,i_/γ_i,k_ is plotted for each of the 20 microbial species in Crockers experiment for two (of ten) carbon resources: mannitol and mannose. The yeild‐to‐depletion ratio varies substantially across species.
**Table D1:** Weighted linear regression of abundance correlation versus *D*, under various filtering conditions.
**Figure D3:** Same as Figure 5 in the main text, but with the leftmost point in the left panel removed.

## Data Availability

All code and data supporting this study are archived at Zenodo and are available at https://doi.org/10.5281/zenodo.17334246.
