# Supplementary material for: Niche Overlap Is Not Enough: Same Overlap, Contrasting Fluctuations
Source: Ecol Lett. 2026 Jun 24;29(6):e70425. doi: 10.1111/ele.70425 (PMC13291768; doi:10.1111/ele.70425)
Supplement: Supplementary file 1 — Supporting Information: A. From consumer‐resource to Lotka‐Volterra. Figure A1: Correlations versus niche overlap. Supporting Information: B Calculating correlations. Figure B1: Comparison of numerical (X) and analytical (O) correlations across 100 independent trials. Top: abundance correlations CR(n 1, n 2). Bottom: growth‐rate correlations CR(r 1, r 2). The close alignment across trials demonstrates the accuracy of the theoretical predictions. Supporting Information: C The Yield‐Depletion Mismatch (YDM) metric. Figure C1: Comparison of the abundance correlation cor(λ) and the YDM parameter D(λ). Both functions exhibit the same qualitative shape, share the same peak value, and cross zero at the same points. Supporting Information: D Empirical Results. Figure D1: Time series of relative abundances (fraction of the total community) across nine transfer cycles for 20 microbial strains in one environment (a fixed carbon mixture). Figure D2: The ratio C = λk,i/γi,k is plotted for each of the 20 microbial species in Crockers experiment for two (of ten) carbon resources: mannitol and mannose. The yeild‐to‐depletion ratio varies substantially across species. Table D1: Weighted linear regression of abundance correlation versus D, under various filtering conditions. Figure D3: Same as Figure 5 in the main text, but with the leftmost point in the left panel removed. [file ELE-29-0-s001.pdf]

Supplementary A
From consumer-resource to Lotka-Volterra

Many mathematical models have been developed to describe deterministic competition between species, with the **Lotka-Volterra (LV)** model emerging as one of the most widely recognized frameworks. The LV model has garnered significant praise for its ability to represent complex ecological systems in a canonical and accessible manner. By capturing the general trends in deterministic ecosystem dynamics, the model elucidates emergent behaviors from a minimal set of key parameters [36, 37].

On the other hand, Lotka-Volterra is an effective model that defines the level of niche overlap between species but does not address the question of the origin of this overlap. As we have seen throughout this paper, the question of correlations depends not only on the niche overlap itself but also on the mechanistic factors it reflects. Therefore, while we wish to implement the Lotka-Volterra model in which niche overlap is well-defined and transparent, we also seek a more fundamental model from which the Lotka-Volterra equations can be derived. For this purpose, we will use a version of MacArthurs consumer-resource model.

Another important aspect is the origin of environmental stochasticity in the system. A stochastic element can be added to the Lotka-Volterra equations, but again, its mechanistic origin is unclear. When we use consumer-resource equations, we introduce the stochasticity through the growth rate of the resource, which in turn affects resource availability and, indirectly, the abundance of the consumer. This makes the entire process much more transparent.

A1. THE LOTKA-VOLTERRA (LV) DYNAMICS

The LV model is governed by the following equation:

$$\frac{dn_i}{dt} = n_i - n_i^2 - n_i \sum_{j \neq i}^{S} \alpha_{i,j} n_j \tag{A1}$$

In this formulation:

- n_i represents the population size of species i,
- S is the number of species,
- The term $n_i - n_i^2$ accounts for intrinsic growth and self-limiting effects (logistic growth),
- The term $-n_i \sum_{j \neq i}^{S} \alpha_{i,j} n_j$ captures inter-species interactions, where $\alpha_{i,j}$ denotes the interaction matrix.

All in all, the nonlinear interactions are reflected in the $\boldsymbol{\alpha}$ matrix, whose elements, in the case of three species competition, are

$$\begin{pmatrix} 1 & \alpha_{12} & \alpha_{13} \\ \alpha_{21} & 1 & \alpha_{23} \\ \alpha_{31} & \alpha_{32} & 1 \end{pmatrix}.$$

Most of the results presented in this paper concern symmetric $\boldsymbol{\alpha}$ matrices ($\alpha_{i,j} = \alpha_{j,i}$; however, as noted in the main text, we observed the same behavior in asymmetric cases, provided the dynamic admits equilibrium states.

A2. THE CONSUMER-RESOURCE (CR) MODEL

Our version of MacArthur consumer-resource model is,

$$\dot{n_i} = -n_i + n_i \sum_{k=1}^{Q} \gamma_{i,k} R_k \tag{A2}$$

$$\dot{R_k} = R_k - R_k^2 - R_k \sum_{i=1}^{S} \lambda_{k,i} n_i \tag{A3}$$

Where:

- R_k is the density of resource k,
- Q is the number of resources,
- $\gamma_{i,k}$ is the yield matrix (how the availability of the resource k is translated into the growth rate of the consumer i),
- $\lambda_{k,i}$ is the depletion matrix (how the presence of the consumer i affects the abundance of the resource k).

A3. DERIVATION OF THE LV MODEL FROM THE CR DYNAMIC

The consumer-resource model is our workhorse in this paper. To derive the corresponding LV from it, one assumes $\dot{R}_k = 0$, yielding,

$$R_k = 1 - \sum_{i=1}^{S} \lambda_{k,i} n_i \tag{A4}$$

Substituting Eq. (A4) into Eq. (A2), we obtain:

$$\dot{n_i} = \left[\sum_{k=1}^{Q} \gamma_{i,k} - 1\right] n_i - n_i^2 \sum_{k=1}^{Q} \gamma_{i,k}\lambda_{k,i} - n_i \sum_{k=1}^{Q} \sum_{\substack{j=1 \\ j\neq i}}^{S} \gamma_{i,k}\lambda_{k,j} n_j \tag{A5}$$

This formulation enables direct comparison between MacArthurs CR model and the LV model (Eq. (A1)) by choosing parameters such that:

$$\sum_{k=1}^{Q} \gamma_{i,k} = 2 \tag{A6}$$

$$\sum_{k=1}^{Q} \gamma_{i,k}\lambda_{k,i} = 1 \tag{A7}$$

$$\sum_{k=1}^{Q} \gamma_{i,k}\lambda_{k,j} = \alpha_{i,j}, \qquad j \neq i. \tag{A8}$$

The justification for the assumption $\dot{R}_k = 0$ can be given in two ways. One is the assumption that the dynamics of the resource are much faster than those of the consumer; hence, the resource lies on a so-called fast manifold and reaches equilibrium before the consumer changes appreciably. However, even if we drop this assumption, as long as an equilibrium state of the system exists, it must satisfy $\dot{R}_k = 0$, and therefore the consumers will obey the effective Lotka–Volterra equations.

Although the derivation of the effective Lotka-Volterra form relies on a quasi-steady-state (QSS) reduction of the resource dynamics, our numerical simulations of the full stochastic consumer-resource system do not assume strict time-scale separation between consumers and resources. In particular, we varied the relative rates of consumer and resource dynamics and found that the qualitative dependence of abundance correlations on the yield-depletion mismatch (D) remains unchanged across regimes. Thus, the decoupling between niche overlap and temporal abundance correlations does not hinge on fast resource dynamics, but reflects the underlying structure of the consumer-resource interactions themselves.

A4. PARAMETER REDUNDANCY

The constraints shown in Eqs. (A6)-(A8) can be formalized using matrix notation. Let $\mathbf{\Gamma}$ be the yield matrix with elements $\gamma_{i,j}$, and $\mathbf{\Lambda}$ be the depletion matrix with elements $\lambda_{j,i}$. Eqs. (A7)-(A8) implies that the interaction matrix $\boldsymbol{\alpha}$ is given by:

$$\boldsymbol{\alpha} = \mathbf{\Gamma}\mathbf{\Lambda},$$

and the linear growth rate of each consumer species is one iff Eqs. (A6) is satisfied, i.e., if each row of $\mathbf{\Gamma}$ is constrained to sum to 2.

This mapping, however, is highly redundant: many different pairs of $\mathbf{\Gamma}, \mathbf{\Lambda}$ matrices yield the same $\boldsymbol{\alpha}$. In the deterministic regime, this redundancy is inconsequential, as the resulting LV dynamics depend only on $\boldsymbol{\alpha}$. But in the presence of environmental noise, the specific structure of $\mathbf{\Gamma}$ and $\mathbf{\Lambda}$ influences how fluctuations propagate through the system, making the choice of parameterization meaningful.

Example (redundant factorizations).

As an example, let us show how two distinct pairs $(\mathbf{\Gamma}^{(1)}, \mathbf{\Lambda}^{(1)})$ and $(\mathbf{\Gamma}^{(2)}, \mathbf{\Lambda}^{(2)})$ both produce the same LV interaction matrix

$$\boldsymbol{\alpha} = \begin{bmatrix} 1 & 3/4 \\ 3/4 & 1 \end{bmatrix}.$$

One can easily verify that both $\mathbf{\Gamma}^{(1)}\mathbf{\Lambda}^{(1)} = \boldsymbol{\alpha}$ and $\mathbf{\Gamma}^{(2)}\mathbf{\Lambda}^{(2)} = \boldsymbol{\alpha}$, where

$$\mathbf{\Gamma}^{(1)} = \begin{bmatrix} 0.7851 & 0.9262 & 0.2888 \\ 0.3204 & 0.8096 & 0.8699 \end{bmatrix}, \quad \mathbf{\Lambda}^{(1)} = \begin{bmatrix} 0.8101 & 0.4616 \\ 0.3061 & 0.1594 \\ 0.2789 & 0.8312 \end{bmatrix},$$
$$\mathbf{\Gamma}^{(2)} = \begin{bmatrix} 0.9419 & 0.5485 & 0.5096 \\ 1.034 & 0.03950 & 0.9264 \end{bmatrix}, \quad \mathbf{\Lambda}^{(2)} = \begin{bmatrix} 0.04548 & 0.04468 \\ 1.083 & 0.3479 \\ 0.7126 & 1.015 \end{bmatrix}. \quad (16)$$

Entries were rounded to four significant figures; each row of $\mathbf{\Gamma}$ sums to 2.

A5. GENERATING YIELD AND DEPLETION MATRICES

Throughout this paper, we fixed the parameter values in the LV model (thereby also determining the niche overlap) and then searched for many CR systems that, in the limit

$\dot{R}_k = 0$, yield the same parameter values, making use of the redundancy. To this end, we employed two methods. Here we provide the details of these two methods.

Sampling Method 1 - Exploring Λ Through Null-Space Perturbations

Each row of Γ was generated by drawing a random vector with positive entries and normalizing it so that the row sums to 2:

$$\vec{\gamma}_i = 2 \cdot \frac{\vec{v}_i}{\sum_j v_{i,j}}, \quad i = 1..S$$

This ensures the constraint (A6).

Given such a Γ, we constructed a compatible Λ by solving:

$$\Gamma\Lambda = \alpha$$

Since Γ is a $S \times Q$ matrix, the solution for Λ is underdetermined. The Moore-Penrose pseudoinverse $\Gamma^{\dagger}$ provides us with a particular solution:

$$\Lambda^0 = \Gamma^{-1}\alpha.$$

To explore the solution space, we added to this particular solution a random component from the null space of Γ, yielding:

$$\Lambda = \Lambda^0 + \epsilon \mathrm{Null}(\Gamma)$$

where ϵ is a random matrix of appropriate dimensions. This method ensures that all generated Λ matrices satisfy the constraints (A6)-(A8) while allowing for variability due to redundancy in the CR-to-LV projection.

Sampling Method 2 - Constrained Optimization Approach

In this method, we used numerical optimization to generate matrices Γ and Λ that approximately satisfy the condition $\Gamma\Lambda \approx \alpha$.

We jointly optimized the entries of $\Gamma \in \mathbb{R}^{S\times Q}$ and $\Lambda \in \mathbb{R}^{Q\times S}$ by minimizing the following cost function:

$$\mathrm{Cost} = \frac{\|\Gamma\Lambda - \alpha\|_{\mathrm{F}}^2}{\|\alpha\|_{\mathrm{F}}^2} + w \cdot \sum_{i=1}^{S} \left(\sum_{j=1}^{Q} \gamma_{ij} - 2 \right)^2$$

Here, the first term is the normalized Frobenius norm quantifying the reconstruction error between the product $\Gamma\Lambda$ and the target interaction matrix α. The second term penalizes deviations from the desired row-sum constraint $\sum_j \gamma_{ij} = 2$, and w is a tunable penalty weight.

The optimization was subject to the hard constraint that all elements of Γ and Λ lie within the interval $[0, 2]$. This was implemented using the Sequential Quadratic Programming (SQP) algorithm within MATLABs `fmincon` solver.

A6. CONSISTENCY BETWEEN THE TWO SAMPLING METHODS

In the main text we pointed out a few trends, like the tendency of abundance correlations to approach (-1) as $\alpha \to 1$, i.e., at the time-averaged neutral point. One could hypothetically attribute such a pattern to imperfect sampling of the underlying parameter space. When working at fixed α, some entries of the Γ and Λ matrices are constrained, while the remaining parameters are randomly sampled. It is difficult to guarantee uniform coverage of the entire feasible matrix space under such constraints.

Nevertheless, we believe the observed trend is genuine. In Fig. A1, we compare the average correlations resulting from two different sampling methods for the matrix space (detailed in Supplementary A). As far as can be judged from these results, the approach to the limit of neutral consumer dynamics (i.e., an $\boldsymbol{\alpha}$ matrix whose all entries equal to one) appears to be associated with strongly anti-correlated abundance fluctuations of consumer species.

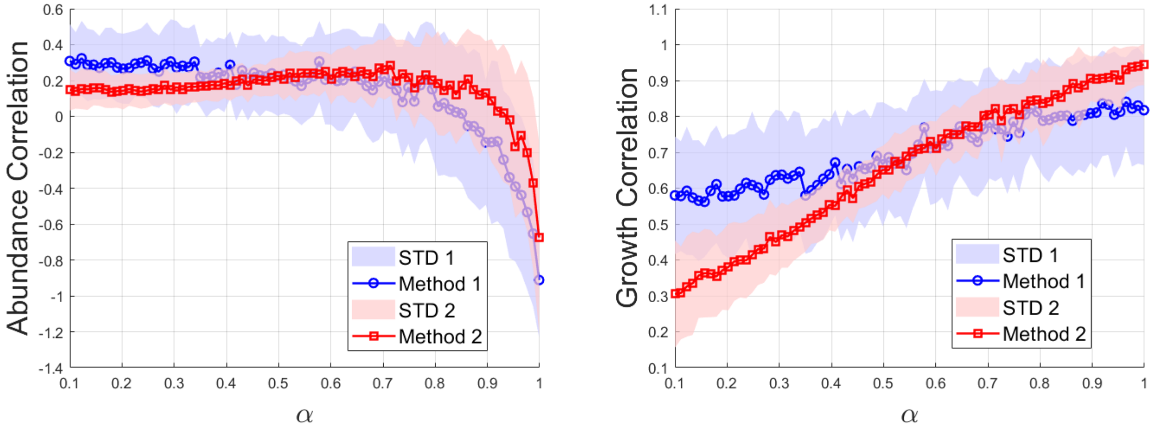

FIG. A1: **Correlations vs. niche overlap.** The mean (markers) and the standard deviation (shaded area) of the correlation values are plotted against niche overlap α for two-species, three-resources dynamics. The correlations in abundance (left panel) are quite a bad indicator for niche overlap - they are almost independent of α until $\alpha > 0.8$, say, and then drop towards (-1) at the "neutral" limit $\alpha \to 1$. On the other hand, the correlations in growth rate (right panel) are roughly linear in niche overlap. The space of Γ and Λ matrices was sampled by the two different methods described above. While these two methods yield slightly different quantitative results, the qualitative picture is quite robust.

Supplementary B
Calculating correlations

To understand how environmental fluctuations propagate through the consumer-resource dynamics and generate correlations in consumer species abundances, we analyze the system near its deterministic equilibrium. Specifically, we linearize the following stochastic differential equations:

$$\frac{dn_i}{dt} = -n_i + n_i \sum_{k=1}^{Q} \gamma_{i,k} R_k, \tag{B1}$$

$$\frac{dR_k}{dt} = R_k \left(1 - R_k - \sum_{i=1}^{S} \lambda_{k,i} n_i \right) + \sigma_k \zeta_k(t) R_k, \tag{B2}$$

where $n_i(t)$ and $R_k(t)$ denote the abundances of consumer i and resource k, respectively. The matrices $\gamma_{i,k}$ and $\lambda_{k,i}$ encode the yield and depletion coefficients, and $\zeta_k(t)$ are independent, zero-mean Gaussian white noise processes, $\langle \zeta_k(t) \zeta_{k'}(t') \rangle = \delta_{k,k'} \delta(t - t')$.

Our goal is to compute the abundance correlations $\langle \delta n_i \delta n_j \rangle$ and the growth rate correlations $\langle \delta r_i \delta r_j \rangle$, for consumer species i and j, at the vicinity of the steady state. In what follows we provide three different methods: a semi-analytic approach, a numerical approach and a full analytic solution for a simple case.

B1. THE SEMI-ANALYTIC APPROACH

We decompose each variable into its deterministic equilibrium value and a stochastic fluctuation:

$$n_i(t) = \bar{n}_i + \delta n_i(t), \tag{B3}$$

$$R_k(t) = \bar{R}_k + \delta R_k(t). \tag{B4}$$

Expanding the dynamics to first order in δn_i and δR_k leads to a linearized system. We

collect all fluctuations into a single vector:

$$\mathbf{x}(t) = \begin{bmatrix} \delta n_1(t) \\ \vdots \\ \delta n_S(t) \\ \delta R_1(t) \\ \vdots \\ \delta R_Q(t) \end{bmatrix} \in \mathbb{R}^{S+Q}. \tag{B5}$$

The linearized stochastic dynamics can now be expressed as:

$$\frac{d\mathbf{x}}{dt} = \mathcal{M}\mathbf{x}(t) + \boldsymbol{\eta}(t), \tag{B6}$$

where $\mathcal{M} \in \mathbb{R}^{(S+Q)\times(S+Q)}$ is the Jacobian matrix evaluated at equilibrium, and $\boldsymbol{\eta}(t)$ encodes the noise terms acting on the system.

A. The structure of stochasticity

The noise is assumed to act only on the resource variables and to be proportional to the equilibrium abundance of each resource. Thus, the noise vector takes the form:

$$\boldsymbol{\eta}(t) = \begin{bmatrix} \mathbf{0}_S \\ \boldsymbol{\zeta}(t) \odot \bar{\mathbf{R}} \end{bmatrix}, \tag{B7}$$

where $\boldsymbol{\zeta}(t) \in \mathbb{R}^Q$ is a vector of independent white noise processes with

$$\langle \zeta_k(t)\zeta_{k'}(t')\rangle = \sigma_k^2 \delta_{kk'}\delta(t-t'),$$

and $\odot$ denotes elementwise multiplication.

The noise covariance matrix is then:

$$\mathbf{W} = \langle \boldsymbol{\eta}(t)\boldsymbol{\eta}^\top(t')\rangle = \delta(t-t') \begin{bmatrix} \mathbf{0}_{S\times S} & \mathbf{0}_{S\times Q} \\ \mathbf{0}_{Q\times S} & \mathrm{diag}(\sigma_1^2\bar{R}_1^2,\ldots,\sigma_Q^2\bar{R}_Q^2) \end{bmatrix}. \tag{B8}$$

B. Abundance Correlations

We denote the steady-state covariance matrix as:

$$\mathbf{C} = \langle \mathbf{x}(t)\mathbf{x}^\top(t)\rangle, \tag{B9}$$

where $\mathbf{x}(t)$ collects both consumer and resource fluctuations. In the steady state, this covariance matrix satisfies the Lyapunov equation:

$$\mathcal{M}\mathbf{C} + \mathbf{C}\mathcal{M}^\top = -\mathbf{W}. \tag{B10}$$

Let $\mathcal{M}$ be diagonalizable, i.e., $\mathcal{M} = \mathbf{P}\Theta\mathbf{T}$ where:

- $\Theta = \mathrm{diag}(\theta_1, \ldots, \theta_{S+Q})$ is the diagonal matrix of eigenvalues,
- $\mathbf{P}, \mathbf{T} \in \mathbb{R}^{(S+Q)\times(S+Q)}$ are the right and left eigenvector matrices satisfying $\mathbf{T}\mathcal{M}\mathbf{P} = \Theta$ and $\mathbf{TP} = \mathbf{I}$.

Then, the covariance between any two components x_m and x_ℓ of the fluctuation vector (consumers or resources) can be written as:

$$\langle \delta x_m \, \delta x_\ell \rangle = \sum_{i=1}^{S+Q} \sum_{j=1}^{S+Q} \sum_{k=1}^{Q} P_{m,i} P_{\ell,j} T_{i,S+k} T_{j,S+k} \, \frac{\sigma_k^2 \bar{R}_k^2}{\theta_i + \theta_j}. \tag{B11}$$

Equation (B11) is a general result: it applies equally to consumer-consumer, resource-resource, and mixed consumer-resource correlations. In particular, the resource-resource covariances $\langle \delta R_i \delta R_j \rangle$ used later in the growth-rate analysis follow directly from this formula.

C. Correlation in Growth Rates

The instantaneous growth rate of species i over a small time interval Δt is defined as:

$$r_i(t) = \frac{1}{\Delta t} \ln\left(\frac{n_i(t+\Delta t)}{n_i(t)}\right) = \frac{\ln n_i(t+\Delta t) - \ln n_i(t)}{\Delta t}. \tag{B12}$$

In the limit $\Delta t \to 0$, this becomes the time derivative of the logarithm of abundance:

$$z_i(t) = \ln n_i(t) \quad \Rightarrow \quad \dot{z}_i(t) = \frac{d}{dt} \ln n_i(t) = \frac{\dot{n}_i(t)}{n_i(t)}. \tag{B13}$$

Thus, we define the instantaneous growth rate as:

$$r_i(t) = \dot{z}_i(t) = \frac{\dot{n}_i(t)}{n_i(t)}. \tag{B14}$$

Linearizing the dynamics around the stable equilibrium and assuming that fluctuations in species growth rates are primarily driven by fluctuations in resource levels, we obtain the approximation:

$$\langle \delta r_m \, \delta r_n \rangle = \sum_{i=1}^{Q} \sum_{j=1}^{Q} \gamma_{mi} \gamma_{nj} \, \langle \delta R_i \delta R_j \rangle, \tag{B15}$$

where:

- γ_{mi} is the entry of the consumption matrix $\boldsymbol{\gamma}$, describing the efficiency with which species m utilizes resource i,

- $\langle \delta R_i \delta R_j \rangle$ is the covariance of resource fluctuations, obtained from Eq. (B11).

This formulation shows how species that depend on similar resources exhibit correlated growth-rate fluctuations. The magnitude and sign of $\langle \delta r_m \delta r_n \rangle$ are shaped both by the overlap in resource preferences (via γ_{mi}) and by the resource covariances determined from the general abundance correlation formula.

Calculating the correlations for both abundance and growth rate can also be done numerically by integrating the stochastic differential equations over time. We compared the numerical and analytical results and found close agreement. This reinforces the robustness of our theoretical framework.

B2. NUMERICAL INTEGRATION OF SDE

We complement the linear response analysis with direct numerical simulations of the full stochastic system. Although we integrate the SDE numerically using the Euler-Maruyama scheme (Itô), we implement the *Stratonovich* interpretation of the multiplicative noise by adding the standard Itô-Stratonovich drift correction. Concretely, for a term of the form $\sigma x \circ dW$ we simulate the equivalent Itô SDE

$$dx = \left[a(x) + \frac{1}{2}\sigma^2 x \right] dt + \sigma x \, dW,$$

which is then integrated by Euler–Maruyama. Applying this approach to Eqs. (B1)-(B2), we implemented the following numerical procedure:

$$\begin{aligned} n_i(t+\Delta t) &= n_i(t) + \Delta t \cdot n_i(t) \left(-1 + \sum_{k=1}^{Q} \gamma_{ik} R_k(t) \right) \\ R_k(t+\Delta t) &= R_k(t) + \Delta t \cdot R_k(t) \left(1 + \sigma_k^2/2 - R_k(t) - \sum_{i=1}^{S} \lambda_{ki} n_i(t) \right) + \sigma_k R_k(t) \sqrt{\Delta t} \cdot \zeta_k(t), \end{aligned} \tag{B16}$$

where $\zeta_k(t)$ are independent Gaussian random variables with mean zero and unit variance.

A. Parameter Values and Their Role

We used the following parameters in our simulations:

- **Total time** $T = 10000$: the number of full time units simulated.
- **Time resolution** $\texttt{steps} = 100$: number of integration steps per time unit, yielding a time step of $\Delta t = 1/\texttt{steps} = 0.01$.
- $\boldsymbol{\sigma}_e = 0.05$: strength of environmental noise acting on the resources.
- $N_\tau = 10$: number of resource substeps per consumer step to enforce faster resource dynamics (see below).

1. Time Scale Separation

To mimic scenarios in which resource dynamics operate on faster timescales than consumer dynamics, we updated resource abundances more frequently. Specifically, within each consumer time step of size Δt, we performed $N_\tau = 10$ substeps where only the resource variables were updated using Eq. (B16). This introduces an effective time scale separation and better approximates the quasi-steady-state assumption often used in analytical reductions. Under such timescale separation, i.e., when resource dynamics are fast relative to consumer dynamics, the mapping from the deterministic consumer-resource model to the Lotka-Volterra equations is exact. As we note in Supplementary A3, our conclusions do not depend on this assumption: we observe the same correlation patterns even when resource dynamics are not faster than consumer dynamics.

2. Measurement of Correlations

After a sufficient transient period, we recorded:

- The time series of species abundances $n_i(t)$.
- The instantaneous growth rates:

$$r_i(t) = \frac{1}{\Delta t} \ln \left(\frac{n_i(t + \Delta t)}{n_i(t)} \right).$$

From these, we computed the Pearson correlation coefficients between all species pairs i, j:

$$C_{ij}^{(\text{abundance})} = \text{corr}\left(\{n_i(t)\}, \{n_j(t)\}\right), \tag{B17}$$

$$C_{ij}^{(\text{growth})} = \text{corr}\left(\{r_i(t)\}, \{r_j(t)\}\right). \tag{B18}$$

Only the post-transient portion of the simulation was used to compute these statistics.

B3. NUMERICAL VALIDATION OF THEORETICAL PREDICTIONS

To validate the analytical framework developed above, we performed numerical simulations of the stochastic consumer–resource system with parameter choice $\alpha = 0.9$. The results confirm that the semi-analytic predictions for both abundance and growth-rate correlations are in excellent agreement with direct numerical integration.

A. Single Run Example

Figure B1 shows the time series of two consumer species (n_1, n_2) and three resources (R_1, R_2, R_3) over a long simulation. The consumers fluctuate around their steady-state abundances while the resources remain stable, subject to noise-induced fluctuations.

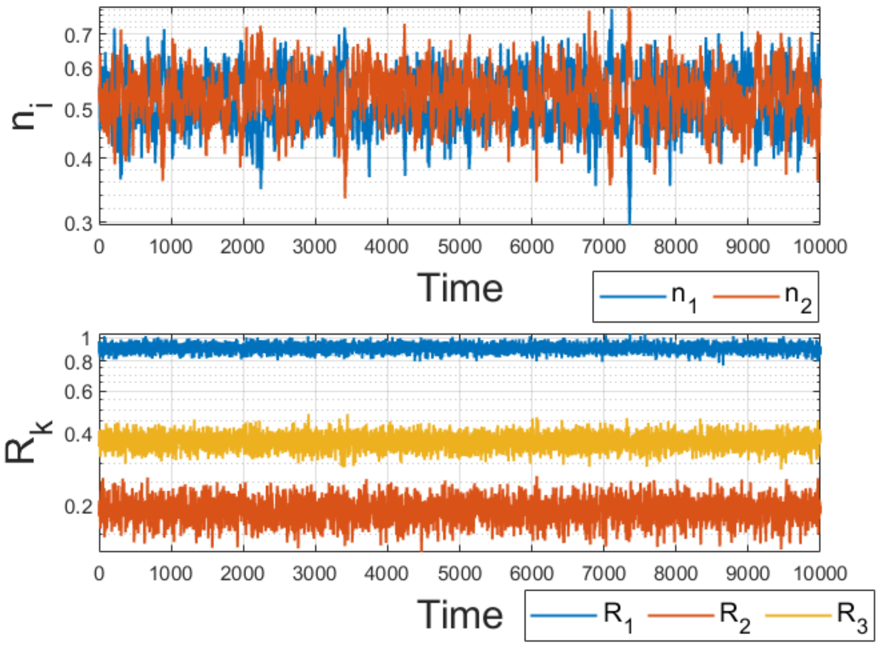

FIG. B1: Time series of consumer abundances (n_1, n_2) and resource abundances (R_1, R_2, R_3) from a numerical integration of the stochastic consumer–resource model with $\alpha = 0.9$.

From these trajectories, we computed correlations and compared them with theoretical

predictions:

$$\begin{aligned}\text{Numerical abundance correlation: } & C^{(\text{num})}_{n_1,n_2} = -0.5722,\\ \text{Theoretical abundance correlation: } & C^{(\text{th})}_{n_1,n_2} = -0.5709,\\ \text{Numerical growth-rate correlation: } & C^{(\text{num})}_{r_1,r_2} = 0.6914,\\ \text{Theoretical growth-rate correlation: } & C^{(\text{th})}_{r_1,r_2} = 0.7035.\end{aligned}$$

The near-perfect agreement illustrates the robustness of the linear response analysis.

B. Ensemble of Trials

To further assess consistency, we repeated the numerical experiment across 100 independent trials with randomized noise realizations. Figure B2 compares the correlations obtained numerically (crosses) with the theoretical predictions (circles). The top panel shows consumer abundance correlations $CR(n_1, n_2)$, while the bottom panel shows growth-rate correlations $CR(r_1, r_2)$.

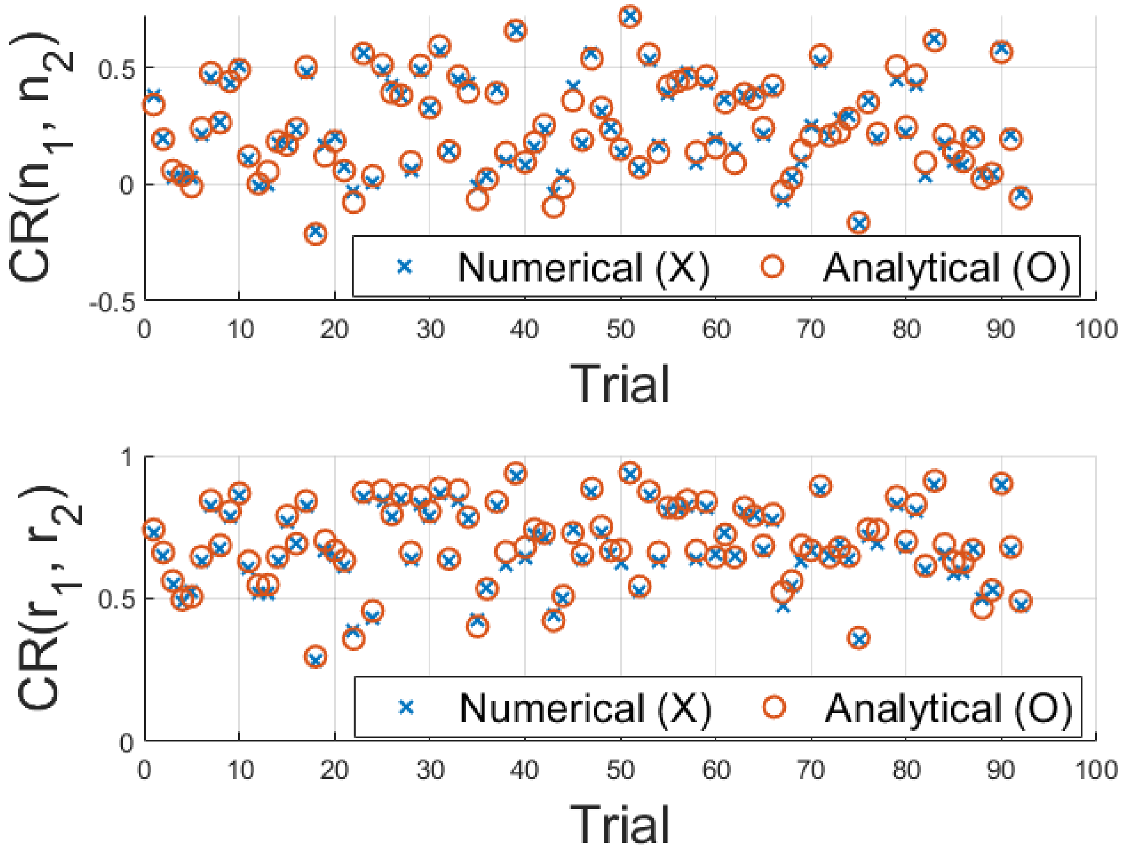

FIG. B2: Comparison of numerical (X) and analytical (O) correlations across 100 independent trials. Top: abundance correlations $CR(n_1, n_2)$. Bottom: growth-rate correlations $CR(r_1, r_2)$. The close alignment across trials demonstrates the accuracy of the theoretical predictions.

Supplementary C

The Yield-Depletion Mismatch (YDM) metric

As shown in the main text, the correlation function is determined by the matrices Γ and Λ. Our aim, therefore, is to identify a clear and tractable metric that captures the key features relevant to the correlation function.

To guide a theory-based conjecture, we first consider a simple illustrative case that admits a transparent analytic solution. Specifically, we select Γ and Λ matrices that generate the effective interaction matrix,

$$\boldsymbol{\alpha} = \begin{bmatrix} 1 & \frac{2}{3} \\ \frac{2}{3} & 1 \end{bmatrix}.$$

We require Γ and Λ such that $\boldsymbol{\alpha} = \Gamma\Lambda$ and each row of Γ sums to 2. To allow for a fully analytic solution, we focus on a system of two species and two resources, with the additional condition that the diagonal elements of Λ are equal. Concretely, the two matrices take the form

$$\Gamma = \begin{bmatrix} \frac{6(2\lambda-1)}{12\lambda-5} & 2 - \frac{6(2\lambda-1)}{12\lambda-5} \\ \frac{4(3\lambda-1)}{12\lambda-5} & 2 - \frac{4(3\lambda-1)}{12\lambda-5} \end{bmatrix}, \qquad \Lambda = \begin{bmatrix} \frac{5}{6} - \lambda & \lambda \\ \lambda & \frac{5}{6} - \lambda \end{bmatrix}.$$

We then calculated the abundance correlation using the method described in Eq. (B11) of Supplementary B. Because the matrices take such a straightforward form, the abundance correlations between the consumer species can be expressed in a concise analytic formula,

$$\mathrm{cor} = 1 - \frac{5}{15 + 12\lambda(6\lambda - 5)}.$$

Next, we calculated the YDM parameter D as described in the main text. For every pair

of species i and j,

$$D \equiv \Delta\lambda - \Delta\gamma$$
$$\Delta\gamma = 1 - \cos\theta_{i,j}(\gamma) \equiv 1 - \frac{\vec{\gamma}_i \cdot \vec{\gamma}_j}{|\vec{\gamma}_i||\vec{\gamma}_j|}$$
$$\Delta\lambda = 1 - \cos\theta_{i,j}(\lambda) \equiv 1 - \frac{\vec{\lambda}_i \cdot \vec{\lambda}_j}{|\vec{\lambda}_i||\vec{\lambda}_j|}.$$

Here, the vector $\vec{\gamma}_i$ is the i-th row of the yield matrix Γ, and $\vec{\lambda}_i$ is the i-th column of the depletion matrix Λ; $|\cdot|$ denotes the Euclidean norm.

For the matrices in our two-species, two-resource system, the explicit expression for D is

$$D = 1 - \frac{1}{26 + 24\lambda(6\lambda - 5)} - \frac{25}{50 + 24\lambda(6\lambda - 5)}.$$

Clearly, cor and D are not the same quantity. Still, a glance at Fig. C1 shows that these two functions share the same qualitative shape, attain the same peak value, and cross zero at identical values of λ. In other words, the YDM parameter provides a faithful estimator for the correlation derived directly from the stochastic dynamics. Importantly, this agreement is not limited to the specific example above: for any choice of α, the peak and zero-crossings of cor and D coincide.

As noted in the main text, another advantage of D is that it is a bounded, normalized metric: since $0 \leq \Delta\gamma, \Delta\lambda \leq 2$, it follows that $-2 \leq D \leq 2$. Consequently, D is scale-free and enables consistent comparisons across systems.

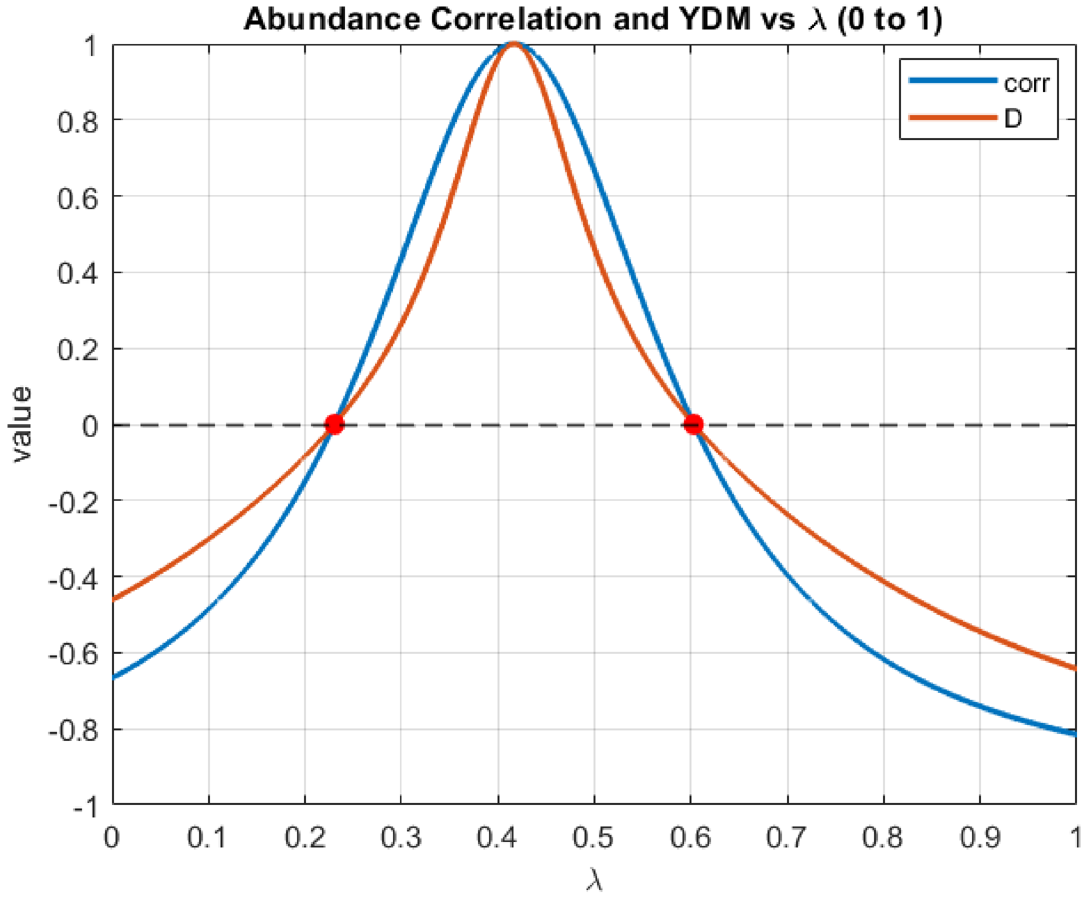

FIG. C1: Comparison of the abundance correlation cor(λ) and the YDM parameter $D(\lambda)$. Both functions exhibit the same qualitative shape, share the same peak value, and cross zero at the same points.

Supplementary D
Empirical Results

D1. DETAILS OF CROCKERS EXPERIMENT

Crocker et al. [5] sought to reduce the complexity of microbial community dynamics by identifying *functional guilds*, groups of strains with similar metabolic traits that respond cohesively to environmental changes. Their central idea was that although microbial communities contain many species, the number of distinct "functional roles" may be far smaller. If these roles can be experimentally identified, community dynamics can be coarse-grained from species level to guild level.

To achieve this, they constructed a synthetic community of 20 soil bacterial strains and characterized each strain in monoculture across 10 carbon sources (arabinose, butyrate, deoxyribose, glucuronic acid, glycerol, mannitol, mannose, melibiose, propionate, and raffinose). From these monocultures they measured:

- the exponential growth rate on each carbon source (via OD measurements),
- the biomass yield per unit carbon and,
- the resource uptake rate, inferred as $r_{i,\alpha} = g_{i,\alpha}/\eta_{i,\alpha}$ from growth and yield.

These measurements provided a growth matrix G describing the metabolic profile of each strain. By clustering rows of G, Crocker et al. defined functional guilds, i.e. sets of strains with overlapping metabolic niches.

The full 20-strain community was then assembled and inoculated into 32 distinct environments, each defined by a random subset of the 10 carbon sources (with total carbon fixed at 25 mM). Each environment was propagated through 9 serial batch cycles of 48 hours growth

followed by a 1:10 dilution into fresh medium. At the end of each cycle, absolute strain abundances were determined using 16S sequencing with a spike-in calibration.

From these experiments Crocker et al. demonstrated that guild cohesion depends on the timescale of environmental fluctuations: on short timescales, strains within a guild fluctuate positively together (shared responses to nutrient pulses), whereas on longer timescales, competitive interactions dominate and abundances of guild members become negatively correlated.

A. Community Time Series: an example

To illustrate the structure of the experimental time-series data, Fig. D1 shows the strain abundances across transfer cycles for a single representative environment. Each curve corresponds to one strain, and its fraction is shown on a logarithmic scale. These trajectories form the basis of the correlation analysis performed in Section D3.

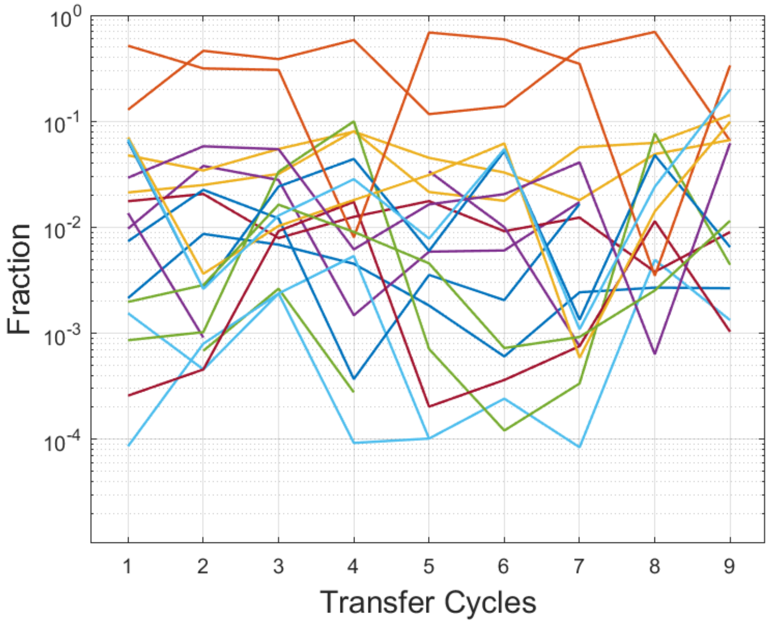

FIG. D1: Time series of relative abundances (fraction of the total community) across nine transfer cycles for 20 microbial strains in one environment (a fixed carbon mixture).

D2. ASYMMETRY BETWEEN YIELD AND DEPLETION

Many models assume a fixed relationship between resource depletion and yield, namely, that the ratio between the amount of resource consumed and the growth rate per unit resource is species-independent. In Figure D2, we plot the yield-to-depletion ratio for two of the carbon resources tested in Crockers monoculture experiments, across all twenty species. The results show that resource-use efficiency varies markedly from one species to another.

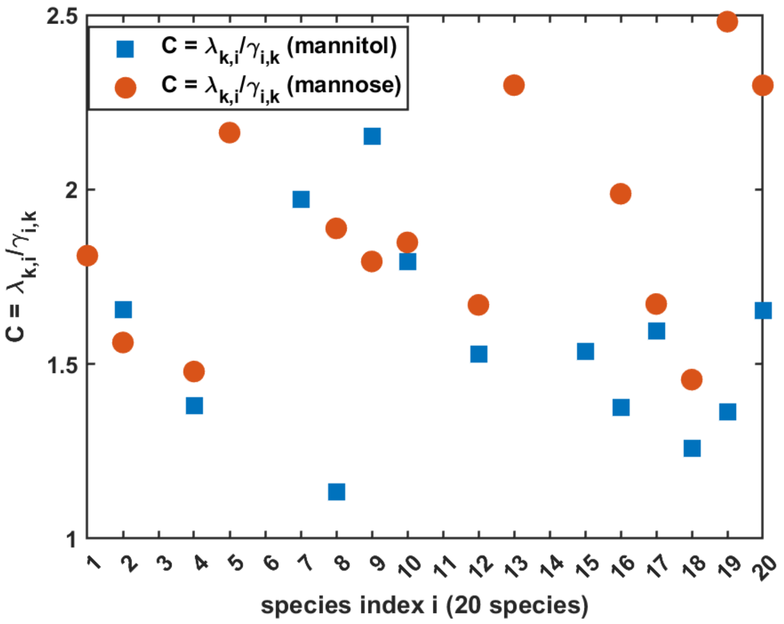

FIG. D2: The ratio $C = \lambda_{k,i}/\gamma_{i,k}$ is plotted for each of the 20 microbial species in Crockers experiment for two (of ten) carbon resources: mannitol and mannose. The yeild-to-depletion ratio varies substantially across species.

D3. USE OF CROCKERS DATASET IN OUR WORK

While Crocker et al. focused on guild-level responses to environmental fluctuation over different timescales, our analysis addressed a different question: whether pairwise abundance correlations track the total functional dissimilarity (D_T) or instead the yield-depletion mismatch (D).

All data used in our analysis were taken directly from the public repository accompanying Crocker et al. For convenience and internal consistency with our notation, we copied the

original files into our own repository (https://doi.org/10.5281/zenodo.19985121.) and renamed them as follows:

- `Gamma.xlsx`: per-capita growth rates of each strain on each carbon source (monoculture measurements),
- `Yield.xlsx`: biomass yields of each strain on each carbon source,
- `strain_abundance_matrix.csv`: - community time series of strain abundances across 9 replicate cycles in 32 environments.

No numerical values were altered; only file names were changed for clarity and consistency with our notation.

We implemented D_T rather than α for the following reason. To calculate niche overlap via the matrix $\boldsymbol{\alpha} = \Gamma\Lambda$, one must rescale the parameters so that the diagonal entries of $\boldsymbol{\alpha}$ are unity. Rather than applying such a normalization, which would require additional assumptions and information beyond the available data, we use D_T as an empirically accessible, coarse measure of overall functional overlap, motivated by the same logic as NO in that it aggregates both depletion and yield differences into a single total dissimilarity score.

A. Construction of Γ and Λ

From the monoculture measurements, we constructed the matrices Γ and Λ as follows. Let $g_{i\alpha}$ denote the reported per-capita growth rate of strain i on resource α, and let $\eta_{i\alpha}$ denote the reported biomass yield. In Crockers formulation, growth decomposes as

$$g_{i\alpha} = r_{i\alpha}\,\eta_{i\alpha},$$

where $r_{i\alpha}$ is the resource uptake rate.

In our notation, we defined

$$\Gamma_{i\alpha} \equiv g_{i\alpha}, \qquad \Lambda_{\alpha i} \equiv r_{i\alpha}.$$

Since uptake rates are not reported directly in the dataset, we reconstructed them elementwise (see details below) via

$$\Lambda_{\alpha i} = \frac{\Gamma_{i\alpha}}{\eta_{i\alpha}}.$$

This gives a depletion matrix Λ consistent with our theoretical formulation.

B. Functional Dissimilarities

For each pair of strains (i, j), we computed cosine dissimilarities in yield and depletion space:

$$\Delta\gamma_{ij} = 1 - \frac{\Gamma_i \cdot \Gamma_j}{\|\Gamma_i\| \, \|\Gamma_j\|},$$

where Γ_i denotes row i of Γ, and

$$\Delta\lambda_{ij} = 1 - \frac{\Lambda_i \cdot \Lambda_j}{\|\Lambda_i\| \, \|\Lambda_j\|},$$

where Λ_i denotes column i of Λ.

From these quantities, we constructed two composite metrics:

$$D_{ij} = \Delta\lambda_{ij} - \Delta\gamma_{ij} \quad \text{(yield-depletion mismatch, YDM)},$$

and

$$D_{T,ij} = \Delta\lambda_{ij} + \Delta\gamma_{ij} \quad \text{(total functional dissimilarity)}.$$

Both metrics were analyzed against abundance correlations in order to test whether correlations are governed by overall functional separation (D_T) or specifically by the asymmetry between depletion and yield profiles (D).

Abundance Correlations

Community abundances were obtained from the time-series dataset. For each of the 32 environments:

1. Strain abundances were normalized to relative abundances (fraction).
2. For each environment, we identified the top N strains by total abundance across transfers. Below, we report results for different values of N.
3. For these strains, we computed pairwise abundance correlations using Spearman's ρ across transfer cycles.

Each pair of strains therefore contributed a data point (D_{ij}, ρ_{ij}) and $(D_{T,ij}, \rho_{ij})$ for each environment in which both strains were among the top N selected.

Averaging and Statistical Analysis

To reduce noise from sparsely sampled strain pairs, identical D (or D_T) values occurring across environments were grouped, and only those represented in at least a specified minimum number of environments (i.e., strain pairs that remained within the top-N across all transfer cycles) were retained. For each retained value, we computed the mean abundance correlation and its standard error. Note that D_{ij} and $D_{T,ij}$ depend only on monoculture measurements and are therefore fixed for each strain pair, whereas abundance correlations were computed separately for each environment.

We then performed weighted linear regression of mean correlation versus D (and separately versus D_T), using inverse standard errors as weights. Statistical significance of the slope was assessed via a two-sided t-test.

While Crocker *et al.* [5] were assessing the possibility of guild-level coarse-graining, here we use the same measurements for a different perpose: to construct explicit yield and depletion profiles and test whether abundance correlations are governed by D_T or specifically by the yield-depletion mismatch D.

D4. ROBUSTNESS ANALYSIS

To test the robustness of our empirical findings, we systematically explored how the relationship between the yield-depletion mismatch parameter D and abundance correlations depends on three filtering parameters:

- `Top N_sp`: Number of top-abundant species per environment included in the analysis (5, 10, or 15)

- `rep_start`: The first batch replicate to include (e.g., starting from replicate 1, 2, or 3 to exclude early-time transients)

- `min_count`: Minimum number of environments in which a pair must co-occur to be included in the average (2, 5, or 10)

We applied these filters within the analysis script, which performs the following steps:

1. Loads species abundance trajectories and monoculture yield/depletion data.

2. For each environment, computes relative abundance time series for the top N most abundant species.

3. Calculates Spearman correlations between all pairs of selected species.

4. Computes the corresponding yield-depletion mismatch $D = \Delta\lambda - \Delta\gamma$ for each pair.

5. Bins data by unique D values (only including those with $\geq$ `min_count` replicates) and computes mean correlation and standard error of the mean (SEM) per bin.

6. Performs a weighted linear regression using 1/SEM as weights to assess the significance of the trend.

D5. ROBUSTNESS ANALYSIS: SUMMARY OF RESULTS

The table below summarizes the regression results for each combination of filtering parameters. For `Top N_sp` = 10 and 15, we observe a consistent, statistically significant positive slope in the relationship between D and abundance correlation, with p-values well below 0.05. In contrast, results for `Top N_sp` = 5 show weaker and nonsignificant trends, due to insufficient sample size and increased noise.

TABLE D1: Weighted linear regression of abundance correlation vs. D, under various filtering conditions.

**Top N**	**Min Count**	**Replicates**	**Fit Equation**	**p-value**
10	2	1–9	$2.5833 \cdot D - 0.0051$	0.00327
10	2	2–9	$2.6915 \cdot D - 0.0290$	0.00132
10	2	3–9	$2.5729 \cdot D + 0.0069$	0.00480
10	5	1–9	$2.5005 \cdot D - 0.0569$	0.00722
10	5	2–9	$2.5624 \cdot D - 0.0656$	0.00581
10	5	3–9	$2.2118 \cdot D - 0.0522$	0.01410
10	10	1–9	$2.5981 \cdot D - 0.0790$	0.01550
10	10	2–9	$2.4302 \cdot D - 0.0820$	0.02890
10	10	3–9	$2.1173 \cdot D - 0.0781$	0.03350
15	2	1–9	$1.3832 \cdot D + 0.0217$	0.00643
15	2	2–9	$1.4242 \cdot D + 0.0164$	0.00605
15	2	3–9	$1.4101 \cdot D + 0.0117$	0.00622
15	5	1–9	$1.3931 \cdot D + 0.0211$	0.00899
15	5	2–9	$1.4449 \cdot D + 0.0141$	0.00548
15	5	3–9	$1.3473 \cdot D + 0.0153$	0.01120
15	10	1–9	$1.3961 \cdot D + 0.0208$	0.00921
15	10	2–9	$1.4475 \cdot D + 0.0139$	0.00566
15	10	3–9	$1.3445 \cdot D + 0.0156$	0.01180
5	2	1–9	$2.4353 \cdot D - 0.2048$	0.32500
5	2	2–9	$2.8804 \cdot D - 0.2195$	0.25400
5	2	3–9	$3.1423 \cdot D - 0.2053$	0.23800
5	5	1–9	$2.7975 \cdot D - 0.2013$	0.35000
5	5	2–9	$3.3280 \cdot D - 0.2274$	0.24600
5	5	3–9	$4.9231 \cdot D - 0.2606$	0.13900
5	10	1–9	$3.1753 \cdot D - 0.3252$	0.38100
5	10	2–9	$4.1148 \cdot D - 0.3683$	0.16900
5	10	3–9	$4.2064 \cdot D - 0.3786$	0.14400

These findings confirm that the predictive power of D is robust across filtering schemes and supports our theoretical prediction that abundance correlations are governed by yield-depletion asymmetry rather than total functional dissimilarity.

D6. ROBUSTNESS ANALYSIS: EFFECT OF THE LEFTMOST POINT

The relationships between pairwise abundance correlations and YDM are shown in the left panel of Fig. 5 in the main text. One may wonder whether the single leftmost data point unduly influences the result. To assess this, we removed that point from the analysis and recalculated the p-values. As shown in Fig. D3, the results remain essentially unchanged. The corresponding p-values are $p = 0.006$ for the YDM (D) and $p = 0.44$ for the total functional dissimilarity D_T.

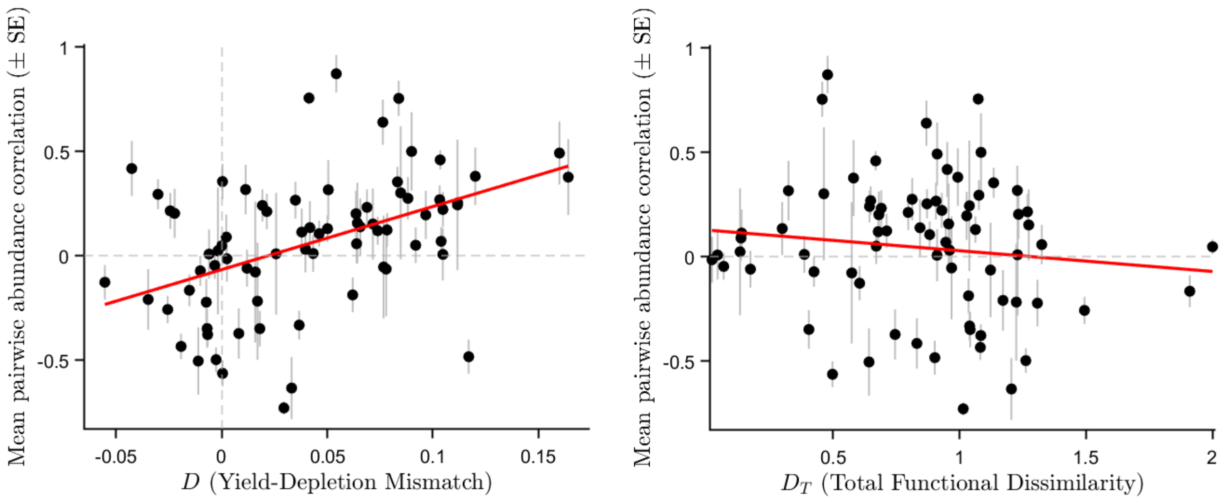

FIG. D3: Same as Fig. 5 in the main text, but with the leftmost point in the left panel removed.
